# Orthopedic Injuries Following the East Azerbaijan Earthquake

**DOI:** 10.5812/traumamon.8322

**Published:** 2013-05-26

**Authors:** Asghar Elmi, Jafar Ganjpour Sales, Ali Tabrizi, Jafar Soleimanpour, Mohammad Ali Mohseni

**Affiliations:** 1 Department of Orthopedics and Trauma Surgery, Shohada Educational Hospital, Tabriz University of Medical Sciences, Tabriz, IR Iran

**Keywords:** Mass Casualty, Earthquakes, Cumulative Trauma Disorders

## Abstract

**Background:**

Evaluating demographic characteristics, distribution and types of orthopedic injuries following major earthquakes may be helpful in future planning for disasters.

**Objectives:**

This study aimed to analyze data from trauma patients with extremity injury resulting from the earthquakes of East Azerbaijan, Iran.

**Patients and Methods:**

Medical records of 686 patients admitted to Shohada hospital, Trauma Center of Tabriz University of Medical Sciences were reviewed. There were 200 patients with extremity injury assessed. Demographic characteristics and patterns of injuries in these patients were evaluated.

**Results:**

In this study, there were 105 females (52.5%) and 95 males (47.5%), out of which, 6 (3%) patients with associated severe head injuries died. The most common sites of injury were lower extremities (81 patients, 41.5% of total victims) while 32 patients (16%) suffered from both upper and lower extremity injuries. Open Fractures were seen in lower extremities of 26 (13%) patients. Compartment syndrome was observed in 3 (1.5%) patients with lower limb fractures. Also, 42 (21%) patients living in Tabriz were injured while they were running away (falling down the stairs and jumping out of windows).

**Conclusions:**

Extremity injuries especially open fractures of lower limbs account for the majority of hospitalized victims. Compartment syndrome is one of the main problems that should be addressed. Our study demonstrates that people still need more education regarding earthquakes and the government should direct more attention to this issue.

## 1. Background 

Earthquakes are one of the most catastrophic natural disasters. Earthquakes can cause a dramatic number of casualties. After the earthquake, health management and treatment centers are confronted with a large number of trauma patients and this causes sudden and serious crisis (1). Only if we know the protocol of earthquakes well, can we allocate the medical resources and implement medical relief operations efficiently (1). Quakes usually occur suddenly with little or no warning and hence, they are often more devastating than other natural disasters (1). Earthquake damage, death toll, managerial protocols, etc. vary in different countries and are influenced by several factors such as the timing of the earthquake (the time during day and night and the day of week) (2). More information is needed about types and profiles of multiple injuries sustained (2). This can add essential information to better plan and more readily adapt the surgical management of the injured, following earthquakes (2). During a summer day on the 11^th^ of August 2012, two large earthquakes hit the East Azerbaijan province of Iran. The magnitudes of these two earthquakes were 6.4 and 6.3 on the Richter scale, respectively.

## 2. Objectives

The present study aims to evaluate the most common post-earthquake musculoskeletal injuries in patients hospitalized at Shohada hospital of the Tabriz University of Medical Sciences as a tertiary referral trauma management center in East Azerbaijan. Demographic characteristics and patterns of injuries of these patients were evaluated.

## 3. Patients and Methods

This descriptive study evaluated all victims of East Azerbaijan earthquakes referred directly from the scene to Shohada Hospital (Trauma Center of Tabriz University of Medical Sciences). The subjects of the study firstly had their medical records evaluated. To better handle the large number of patients admitted within an hour of the quake, all residents were summoned to the center. Four first-year orthopedic residents triaged patients according to their clinical conditions. They were divided into three groups (outpatient, hospitalized, and CPR patients). Overall, 686 patients were visited at the emergency department, out of which, 200 patients had injuries on their extremities with or without any associated injuries in other organs. To calculate an ISS for an injured person, the body is divided into 6 ISS (injury severity score) body regions, which are: head and neck (including cervical spine); face (including the facial skeleton, nose, mouth, eyes and ears); chest (including thoracic spine and diaphragm); abdomen or pelvic area (including abdominal organs and lumbar spine); extremities or pelvic girdle (including pelvic skeleton); and external (skin). Each injury in the body region is ranked according to the Abbreviated Injury Scale (AIS). AIS classifies each injury according to its relative severity on a 6 ordinal scale: 1 (minor), 2 (moderate), 3 (serious), 4 (severe), 5 (critical), and 6 [maximal (currently untreatable)]. In this study, ISS (Injury Severity Score) was calculated for the victims using an online software (www.trauma.org). The ISS score takes values from zero to 75. The study was approved by the Ethics Committee of Tabriz University of Medical sciences.

### 3.1. Statistical Analysis

All data was statistically analyzed using SPSS-16 software (Statistical Package for the Social Sciences, SPSS Inc, Chicago, Il, USA). Continuous variables were shown as mean ± standard deviation. Independent t-test and Chi-square statistical test were used to evaluate qualitative and quantitative variables, respectively. In this study, P ≤ 0.05 was regarded as statistically significant.

## 4. Results

**Figure 1.**
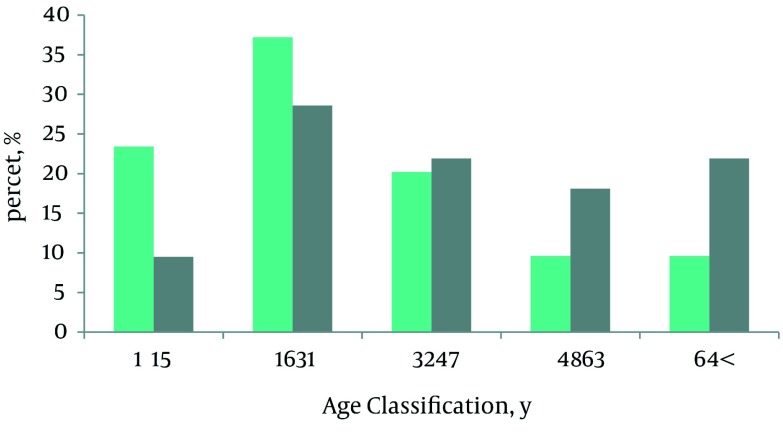
Age and Gender Distribution of Traumatic Patients Following the East Azerbaijan Earthquake

In total, 200 victims with extremity injuries were evaluated and 81.4% of them were hospitalized during the first hours after the earthquake. CPR was performed for 6 (3%) patients but unfortunately all six patients died. They had extremity fractures with severe head injury and they were assigned an ISS score of 75. Average ISS calculated for victims was 20.5 ± 11.5; 36 (18%). There was no difference between the two genders considering severity scores. The details of AIS (Abbreviated Injury Scale) of 6 ISS categories of earthquake victims are shown in Table 1. There were 42 (21%) patients living in Tabriz whom suffered trauma while fleeing (falling down stairs and jumping out of windows). Others were referred from the earthquake-stricken areas after evacuation. Demographic characteristics of the patients are shown in Table 2. There was a significant difference between male and female victims with regard to age (P < 0.001) but there was no difference between them with regard to gender. Figure 1 refers to age and gender distribution of the patients. The more common sites of injury were the extremities (184 patients, 92% of all victims) including upper extremities in 71 cases (35.5%) and lower extremities in 81 cases (40.5%); and 32 patients (16%) suffered from both upper and lower extremity injury. Injury to other organs (the abdomen and chest) associated with orthopedic injuries were seen in 36 (18%) patients. Types of injury are detailed in Table 3. Other injuries, not mentioned in Table 1, included closed upper and lower extremity fractures, which were seen in 14 (7%) and 25 (12.5%) patients respectively.

**Table 1.** Details of AIS of 6 ISS categories in Earthquake Victims

AIS: Abbreviated Injury Scale	Head and Neck, No. (%)	Face, No. (%)	Chest, No. (%)	Abdomen, No. (%)	Extremity, No. (%)	External, No. (%)
**1- Minor**	11 (5.5)	8 (4)	5 (2.5)	9 (4.5)	45 (25.5)	200 (100)
**2- Moderate**	-	-	5 (2.5)	4 (2)	27 (13.5)	-
**3- Serious**	-	-	6 (3)	7 (3.5)	70 (35)	-
**4- Severe**	2 (1)	-	-	-	40 (20)	-
**5- Critical**	4 (2)	-	-	-	18 (9)	-
**6- Maximal**	-	-	-	-	-	-

**Table 2.** Demographic Characteristics of the Patients

Variable	Number of Patients, n = 200
**Gender, Male/Female, No. (%)**	95 (47.5)/105 (52.5)
**Age,Mean± SD, y**	37.7 ± 21.4
**Age Range of patients,y**	3 - 93
**Age of Male Patients,Mean± SD,y**	31.9 ± 19.6
**Age of Female Patients,Mean± SD,y**	42.8 ± 21.6

**Table 3.** Details of the Types of Injuries in Trauma Patients Following the East Azerbaijan Earthquake

Variable	Upper Extremity, No. (%)	Lower Extremity, No. (%)	Total, No. (%)
**Crush syndrome**	8 (4)	4 (2)	12 (6)
**Compartment syndrome**	0	3 (1.5)	3 (1.5)
**Dislocation**	9 (4.5)	5 (2.5)	14 (7)
**Amputation**	6 (3)	2 (1)	9 (4.5)
**Open Fractures**	8 (4)	26 (13)	34 (17)
**Nerve injury**	0	4 (2)	4 (2)
**Spinal fractures**	-	-	11 (5.5)
**Multiple Fractures**	-	-	32 (16)
**Foreign body**	3 (1.5)	7 (3.5)	10 (5)
**Tendon Rupture**	8 (4)	0	8 (4)
**Laceration**	15 (7.5)	3 (1.5)	18 (9)

## 5. Discussion

During the past 20 years, natural disasters have claimed more than 3 million lives worldwide, affected at least 800 million people, and resulted in property damage exceeding $500 billion (3, 4). Earthquakes are one of the most catastrophic natural disasters (5). In East Azerbaijan, the earthquake hit during a summer evening; most victims were elderly, females, and children. Since a majority of youth work on farms, they were not at home and hence were less affected. This was in contrary to Bam earthquake which hit at 5:26 AM (Iran Standard Time) (6, 7) in which, the number of deaths were more than the injured cases. Several studies have emphasized the role of timing in the type of damages and numbers of deaths resulting from earthquakes (2, 6). The mean age of patients from the Azerbaijan earthquakes was 37.7 years old while this was 29.3 years for the Bam disaster. Also, older women made up a greater percentage of victims. This is much different from results of other studies conducted on victims of other earthquakes. In a study on victims of the Northridge earthquake (California, 1994) Peek-Asa et al. compared 0-19 year-old victims with 60-79 year-old and concluded that the aged ones suffered from earthquake-related injuries 10.9 times more than the younger ones. They suggested that the older the people, the dramatically higher the rate of earthquake-related injuries (8). Also in the study conducted by Kuwagata et al. on some of the Hanshin-Awaji earthquakes (Japan, 1995), it was made clear that most victims fell between 40-70 years of age (9). Like the Bam earthquake, generally, there was no significant difference between the victims considering the percentages of males and females. But a significant difference was seen considering the mean age of the victims (7). Our study was also in accordance with some previous studies demonstrating that there was no significant difference between proportions of men and women injured in earthquakes. Mohebbi et al. reported that there were 467 men (54.7%) and 387 women (45.3%) injured in the Bam earthquake 2003. According to the report by Peek-Asa et al., however, the Bam earthquake (10) which occurred in 2003 demonstrated that women were more vulnerable than men to earthquake injuries with a 2.4 times higher injury risk (8). In our study, older women were more likely to be injured. Probably, the lower age of our patients may relate to factors such as the predominance of a young population in Iran especially in small cities and the high intensity of the disasters making the elderly unable to immediately flee from the scene. Appropriate triage of the victims is one of the major issues during the crisis (2). In the Bam disaster, the victims were triaged during the evening following the tragedy because the field hospitals were not established until the next day and two major hospitals of Bam were destroyed (2). In the Azerbaijan earthquakes, the mortality rate was reduced due to close proximity of the medical center to the earthquake-stricken areas and the appropriate triaging of the victims. Extremity injuries were the most common post-earthquake trauma (7). Following the Bam earthquake, the study conducted by Salimi et al. referred to 274 patients suffering from extremity injuries (7). Another study by Lu-Ping et al. in China reported that in 3177 injuries, 1476 (46.5%) had fractures, and 108 (12.6%) had fractures associated with crush and soft tissue injuries (5). In another report from the Hanshin-Awaj earthquake by Ukai, 20 out of 76 victims (26.3%) suffered from extremity injuries (11). Moreover, in the report presented by Peek-Asa et al. analyzing 138 survived hospitalized victims of Northridge earthquake, it was shown that there were 74 (53.6%) and 26 (18.8%) patients with lower and upper extremities injury, respectively (8). As seen, extremity injuries are common in earthquakes thus other related complications such as compartment syndrome and, therefore, probability of death increase. Our study demonstrated a 1.5% incidence rate of compartment syndrome, which is one of the major problems that should be considered. According to previous studies conducted following earthquakes, musculoskeletal injuries distribution was higher than that of other organs. Mulvey et al. (12) and Bulut et al. (13) reported that limb injuries account for 50 - 66% of the total number of injuries incurred during earthquakes. Fractures of the lower extremities were seen more often than those of the upper extremities. Similar to previous reports, in our study prevalence of fractures was higher in lower limbs.

In spite of the lack of damage to buildings of Tabriz city during the Azerbaijan earthquake, injuries and fractures of the Tabriz population (21%) was notable. Extremity injuries especially open fractures of lower limbs account for the majority of hospitalized victims. Most fractures occurred as a result of jumping or falling down stairs. Therefore, people need to be educated on escape tactics.
